# The Effect of Exercise‐Induced Muscle Damage on Lower Limb Side Cut Biomechanics and Task Achievement in Male and Female Team Sport Athletes

**DOI:** 10.1002/ejsc.70051

**Published:** 2025-08-30

**Authors:** Chelsea L. Oxendale, Jamie Highton, Craig Twist, Grace Smith

**Affiliations:** ^1^ Division of Public Health, Sport and Wellbeing University of Chester Chester UK; ^2^ School of Sport and Exercise Sciences Liverpool John Moores University Liverpool UK

**Keywords:** 3D analysis, biomechanics, exercise, recovery, team sport

## Abstract

The effect of exercise‐induced muscle damage (EIMD) on three‐dimensional side cut biomechanics and task achievement were assessed in 16 team sport athletes (eight males and eight females) who completed 45° side cuts before and 48 h after multidirectional running. Angular displacement and joint moments of the hip and knee, and GRF impulse (IGRF) during five successful trials of a 45° side cut, were collected using a 3D motion capture system and force platform at both timepoints. At 48 h, participants had more knee internal rotation (*p* = 0.009), knee abduction ROM (*p* = 0.002), lower peak knee extensor moment (*p* = 0.001) and a higher hip‐knee extensor ratio moment (*p* = 0.020). Large increases in IGRF at 48 h in females (*d*; ± 95% CI: 1.4; ± 1.4, *p* = 0.037) suggest a less effective deceleration capacity. Whilst EIMD had no effect on side cut task achievement and sagittal kinematics, EIMD caused participants to shift the extensor demands away from the knee and towards the hip to decelerate the body. Practitioners should be mindful of potential increases in frontal and transverse motions at the knee for athletes with EIMD, which might have implications for strategies to reduce injury risk.

## Highlights

- Multidirectional running mimicking the movement demands of team sport activity offers an ecologically valid model to study EIMD.
- Whilst task achievement and sagittal side cut kinematics were not affected by EIMD, some impairment to transverse and frontal knee kinematics during a side cut were observed, which could have implications for ACL injury risk.
- EIMD caused participants to shift the extensor demands away from the knee and towards the hip during side cutting and caused a less effective deceleration capacity in females.

## 1 Introduction

Symptoms of exercise‐induced muscle damage (EIMD) are common in team sport athletes and persist for up to several days after exercise (Nedelec et al. 2014). Specifically, increases in creatine kinase activity, perceived muscle soreness, and reductions in neuromuscular function and sprint performance have been reported 24–48 h after team sport activity (Nedelec et al. 2014; Oxendale et al. 2016; Snyder et al. 2019). These indirect markers of EIMD are moderately correlated with actions in team sports that occur at pivotal moments in a match (Martínez‐Hernández et al. 2023), such as the number of sprints (*r* = 0.39–0.76) (Nedelec et al. 2014; Oxendale et al. 2016) and accelerations/decelerations athletes perform (*r* = 0.44–0.48) (Oxendale et al. 2016). These data suggest changes of direction, which incorporate a deceleration from a sprint, change of movement direction and acceleration, place a high mechanical strain on the active musculature. Decelerations elicit eccentric braking force requirements which can cause EIMD and reduced co‐ordinated movement proficiency (McBurnie et al. 2022). A reduction in knee proprioception (Torres et al. 2010) and reflex sensitivity (Avela and Komi 1998) have also been reported with EIMD. Such findings might have implications for injury risk, given that a reduction in proprioception has been prospectively shown to increase the odds ratio of knee injury in females (Zazulak et al. 2007). This is especially relevant during congested fixture/training schedules (Dupont et al. 2010), when symptoms of EIMD might be more prevalent for athletes.

High‐intensity decelerations (Oxendale et al. 2016) and side‐cuts (Martínez‐Hernández et al. 2023) are typical movements of team sport athletes. A particular concern associated with these movements is injury to the anterior cruciate ligament (ACL), with increased incidence rates (Maniar et al. 2022) and high injury burden (Awwad et al. 2019). ACL injury incidence in amateur players is higher during preseason (Mouton et al. 2022), which might be associated with intensified training, increases in EIMD and reductions in muscle force. Indeed, decrements in knee flexor strength have been reported in the days after team sport activity (Snyder et al. 2019) and might pose a risk factor for ACL injury (Myer et al. 2009), as the hamstrings play a crucial role in stabilising the knee joint as it experiences frontal and transverse moments (Besier et al. 2003a). Understanding how exercise which mimics team sport activity might influence both the performance and biomechanics of a side cut would support recommendations in the assessment of an athlete's ACL injury risk (Nedergaard et al. 2020). Specifically, when assessing side cut biomechanics, it is essential to consider performance, or task achievement, given that a faster side cut conflicts with safer biomechanics (Dos’Santos et al. 2021).

Whilst some studies have explored the effect of EIMD on walking and running biomechanics (Paquette et al. 2017; Paschalis et al. 2007; Tsatalas, Giakas, Spyropoulos, Sideris, Kotzamanidis, et al. 2013; Tsatalas, Giakas, Spyropoulos, Sideris, Lazaridis, et al. 2013), to the authors' knowledge only two studies have explored the effect of EIMD on landing and side cut biomechanics (Snyder et al. 2019; Tsatalas et al. 2021). Snyder et al. (2019) reported posterior GRF and anterior tibial shear force during a side cut was increased in females after match‐play, when knee isometric strength was decreased. Given the ACL plays a key role in resisting anterior tibial shear force (Butler et al. 1980), these data suggest EIMD could increase ACL injury risk. Conversely, Tsatalas et al. (2021) reported increased peak hip and knee flexion angles and decreased peak knee extensor moment and vertical GRF with symptoms of EIMD, which suggest a protective mechanism to minimise stress on the ACL. While these studies offer some insight to the potential effects of EIMD on ACL injury risk, the use of single joint, low angular velocity EIMD protocols (Paquette et al. 2017; Tsatalas, Giakas, Spyropoulos, Sideris, Kotzamanidis, et al. 2013; Tsatalas et al. 2021) do not mimic the movement patterns of team sport athletes and lack ecological validity, thus further work is needed to explore biomechanical risk factors. For example, Snyder et al. (2019) only assessed females and did not assess knee abduction and internal rotation angles and moments, which are considered key mechanisms of ACL strain and injury (Myer et al. 2015; Navacchia et al. 2019). Moreover, the use of a drop jump (Tsatalas et al. 2021) places less frontal loading on the knee compared with a side cut (Kristianslund and Krosshaug 2013), providing a sub‐optimal assessment of ACL injury risk. These studies also fail to account for the effect of sex, as males and females exhibit distinct knee moment responses during a side cut when fatigued. Specifically, males have displayed increased knee extensor moments (Savage et al. 2018) whilst females demonstrate increased knee adductor moments (Tsai et al. 2009), and an interaction effect between sex and fatigue has previously been reported (Iguchi et al. 2014). Accordingly, it seems prudent to explore the effect of EIMD symptoms on side cut biomechanics in male and female team sport athletes after an ecologically valid model of exercise, such as multidirectional running. Thus, the purpose of the study was to assess the symptoms of EIMD elicited from multidirectional running and its effect on task achievement and side‐cut biomechanics; specifically, tri‐planar hip and knee kinematics and kinetics and GRF impulse. A secondary aim was to assess if EIMD‐induced alterations in side‐cut biomechanics was different between males and females. It was hypothesized that there would be significant main effects for EIMD on side cut biomechanics and interaction effects between sex and EIMD. Specifically, the presence of EIMD was expected to result in a more cautious side cut technique, characterised by increased sagittal joint angles and reduced sagittal joint moments. However, compensatory increases in frontal and transverse joint angles and moments were also anticipated, particularly in females.

## 2 Materials & Method

### 2.1 Participants

With institutional ethics approval from the University of Chester Faculty of Medicine and Life Sciences Research Ethics Committee (1216‐16‐CO‐SES) and adhering to the Declaration of Helsinki, 16 team sport athletes, including 8 males (stature: 175.5 ± 8.0 cm; body mass: 74.6 ± 8.5 kg; age: 21.6 ± 2.2 years) and 8 females (stature: 166.0 ± 8.4 cm; body mass: 60.6 ± 9.2 kg; age: 21.8 ± 3.1 years) were recruited from university standard team sports (football, netball, hockey and rugby). All participants took part in team sport training sessions at least twice per week. To ensure an adequate sample size for repeated measures comparisons (i.e., changes over time within groups associated with EIMD), an a priori sample size calculation based on estimated power and an effect size (ES) reported from a similar study was used. Specifically, effect sizes of 1.49 and 1.00 have been observed for changes in peak GRF and peak knee extension moment with symptoms of EIMD (Snyder et al. 2019; Tsatalas et al. 2021). The smallest effect size (1.00) was used in the a priori sample size calculation using G Power software (power = 0.95, *α* = 0.05) and yielded a sample of 16 to assess the main aim of the study. Participants completed pre‐test health screening to ensure they had no previous history of knee surgery and/or no lower limb injuries in the past 6 months. Participants were also required to refrain from exercise 48 h before each visit.

### 2.2 Design

Participants completed two visits on separate days. In the first visit, baseline measurements of creatine kinase (CK) concentration, perceived muscle soreness, 20 m sprint time and side cut biomechanics were measured. Thereafter, participants completed the multidirectional running trial. In the second visit, repeated measurements of CK concentration, perceived muscle soreness, 20 m sprint time and side cut biomechanics were taken 48 ± 2 h after the multidirectional trial. A single timepoint at 48 h was select given that the symptoms of EIMD typically peak between 24 and 48 h (Howatson and Milak 2009; Nedelec et al. 2014).

### 2.3 Procedures

#### 2.3.1 Multidirectional Trial

Participants completed a warm‐up consisting of two sub‐maximal bouts of the multidirectional trial for habituation, followed by three progressive accelerations over 10 m. The multidirectional trial comprised 12 bouts of ∼60 s of work followed by 120 s of passive rest, where participants covered 175 m and performed 19 changes of direction during each bout. Briefly, participants performed several forward sprint efforts over 10–35 m, interspersed with changes of direction and forward, lateral and backwards jogging. The specific pattern of activity has previously been detailed elsewhere (Oxendale et al. 2017).

#### 2.3.2 CK Concentration

CK concentration was determined from a fingertip capillary sample. A 30 μL sample of whole blood was taken using a spring‐loaded disposable lancet and analysed using a colourimetric assay procedure (Reflotron, Type 4, Boehringer, Germany).

#### 2.3.3 Perceived Muscle Soreness

Participants provided a rating of their perceived muscle soreness for the lower limbs using a visual analogue scale. The sliding scale was numbered on the reserve side, where 0 indicated ‘no soreness on movement’ and 10 indicated ‘muscles too sore to move’. All participants performed a squat to an approximate knee angle of 90° with their hands‐on hips, and then provided a rating of their perceived muscle soreness using the sliding scale.

#### 2.3.4 Sprint Performance

Participants performed three single maximal 20 m sprints on an outdoor running track, interspersed with a 2 min passive recovery. Sprint times were recorded using infrared timing gates (Brower Speed Trap 2; Brower, UT, USA) positioned at the start and 20 m, with the fastest sprint time used for analysis.

#### 2.3.5 Three‐Dimensional Motion Capture of 45° Side Cuts

Twenty‐eight reflective markers were placed on the trunk, pelvis and lower extremities in accordance with the Liverpool John Moores University lower‐limb and trunk model (Vanrenterghem et al. 2010). Thereafter a 3‐s static calibration, and a dynamic calibration for the right and left hip and knee was recorded, to calculate functional joint centres using Visual3D (version 5.1, C‐Motion Inc., Rockville, USA) and in‐built algorithms (Schwartz and Rozumalski 2005). After several practice attempts as part of the warm‐up, participants performed five 45° side cuts on the preferred leg, interspersed with a 1‐min passive recovery. The preferred leg was determined by asking participants which leg they would use to kick a ball with (van Melick et al. 2017). The approach speed was 4–4.5 m·s^−1^ which was assessed using two infrared timing gates (Brower Speed Trap 2; Brower, UT, USA) located 1.5 m apart before the force platform. Successful trials were those in which the approach speed was maintained, the entire foot contacted the force platform and the side cut was performed at the correct exit angle (45°). Three‐dimensional marker trajectories were recorded using seven three‐dimensional cameras (Oqus 7, Qualisys, Sweden) sampling at 300 Hz. Simultaneous measurements of three‐dimensional GRF were recorded on a force platform (Kistler, Switzerland), sampling at 900 Hz. All kinematic and kinetic data were captured using biomechanical software (Qualisys Track Manager 2.15, Qualisys, Sweden).

### 2.4 Data Analysis of 45° Side Cuts

Kinematic and kinetic calculations from tracked marker trajectories and GRF data were conducted using Visual3D (version 5.1, C‐Motion Inc., Rockville, USA). Trajectory data and GRF data were filtered using a fourth‐order, zero‐lag low‐pass Butterworth filter at 12 and 40 Hz, respectively (Collins et al. 2016; Roewer et al. 2014). Angular displacement (°) in all three planes were calculated using Cardan x‐y‐z rotation sequence. The hip and knee angle were measured relative to the next most proximal segment.

Task achievement was evaluated using the first derivative of centre of mass (COM) position to calculate horizontal (resultant of anterior‐posterior and mediolateral) COM velocity at initial contact (IC) and toe off (TO) (Dos’Santos et al. 2021). Trigonometry using anterior‐posterior and mediolateral velocities was then used to calculate the change in COM angle between IC and to confirm the cut angle performed. Three‐dimensional hip and knee internal moments were calculated using Newton‐Euler inverse dynamics procedures. The net internal joint moments produced by joints and ligaments are presented which counterbalance the external joint moments. The joint co‐ordinate system was used as a reference frame for joint moments and all joint moments were normalized to body mass (N·m·kg^−1^). All data were time‐normalized to 100% of stance, which was defined as the time vertical GRF exceeded 50 N. Peak data during the weight acceptance phase of the side cut; defined as the interval between initial contact and peak knee flexion were quantified. To quantify the relative contributions of the hip and knee moments, a peak hip to knee extensor moment (peak hip extensor moment divided by peak knee extensor moment) was calculated (Sigward et al. 2018). Angle data at initial contact were also quantified and are provided as a Supporting Information S1. Vertical GRF impulse (IGRF) during the weight acceptance phase was derived via integration of the vertical GRF over time, normalized to body weight.

### 2.5 Statistical Analysis

Descriptive data are reported as mean ± standard deviation. Normality was assessed using a Shapiro‐Wilk test and the assumptions of parametric statistical analysis were met. Tri‐planar hip and knee angular and moment data and IGRF were analysed using separate two‐way mixed ANOVAs with time as a within‐subject factor, and sex as a between‐subject factor. *p* values were reported for all analyses. Further analyses consisting of effect sizes with accompanying 95% confidence intervals (*d*; ± 95% CI) were performed. The effect size was calculated as the difference in means divided by the pooled standard deviation. An effect size of 0.2, 0.6, and 1.2 were considered small, moderate and large, respectively. All statistical analyses were performed using the Statistical Package for Social Sciences (SPSS, version 22; SPSS Inc., USA) and Microsoft Excel.

## 3 Results

Indirect markers of EIMD and side cut task achievement before and 48 h after multidirectional running are displayed in Table 1 and sex specific data are presented in Supporting Information S1: supplementary file 1. There was a large increase in CK concentration (*F* = 9.261, *p* = 0.009) and perceived muscle soreness (*F* = 82.283, *p* < 0.001) and a moderate increase in 20 m sprint time (*F* = 30.655, *p* < 0.001) 48 h after multidirectional running. No interactions of time and sex were noted for any indirect markers of EIMD. COM cut velocity was similar at baseline and 48 h at IC (*F* = 0.036, *p* = 0.853) and TO (*F* = 0.238, *p* = 0.663). COM cut angle (*F* = 0.041, *p* = 0.842) and stance time (*F* = 0.669, *p* = 0.427) also remained similar 48 h after multidirectional running. An interaction of time and sex was noted for percentage of stance time in weight acceptance (*F* = 8.335, *p* = 0.012). Specifically, females demonstrated a moderate increase in percentage of stance time in weight acceptance (56.0 ± 7.2 cf. 63.1 ± 10.5%; 0.87; ± 0.75) whereas males displayed a small decrease (47.7 ± 4.2 cf. 46.4 ± 4.6%; −0.29; ± 0.66) 48 h after multidirectional running.

**TABLE 1:** In‐direct markers of EIMD before and 48 h after the multidirectional trial.

	Baseline (*n* = 16)	48 h (*n* = 16)	*d*; ± 95% CI
Indirect markers of EIMD			
CK Concentration (U·L^1^)	124.2 ± 52.8	295.6 ± 228.6^a^	3.08; ± 2.13
Perceived soreness (AU)	0.6 ± 0.7	4.3 ± 1.6^a^	4.94; ± 1.15
20 m sprint time (s)	3.3 ± 0.3	3.5 ± 0.3^a^	0.77; ± 0.30
Side cut task achievement			
Centre of mass velocity at IC (m·s^−1^)	4.2 ± 0.2	4.2 ± 0.2	0.04; ± 0.43
Centre of mass velocity at TO (m·s^−1^)	3.8 ± 0.3	3.8 ± 0.2	−0.09; ± 0.42
Change in centre of mass angle from IC to TO (°)	23.3 ± 4.7	23.2 ± 3.6	−0.03; ± 0.35
Stance time (s)	0.23 ± 0.04	0.23 ± 0.04	0.12; ± 0.30
Weight acceptance percentage of stance (%)	51.9 ± 7.1	54.7 ± 11.6	0.38; ± 0.50

Abbreviations: IC = initial contact; TO = toe off.

^a^ indicates a significant difference at 48 h compared to baseline.

### 3.1 Kinematics

**FIGURE 1:**
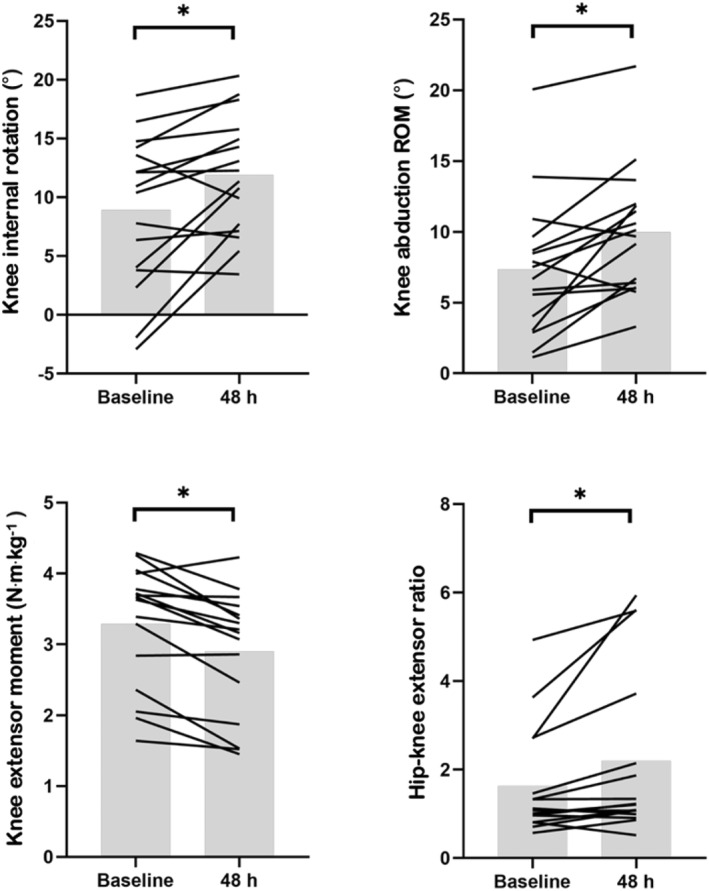
Mean and individual knee internal rotation angle, knee abduction ROM, knee extensor moment and hip‐knee extensor ratio at baseline and 48 h. *indicates a significant difference.

Peak angular data are presented in Table 2 and sex specific peak angular data are presented in Supporting Information S1: supplementary file 2. Peak knee internal rotation angle was higher 48 h after multidirectional running (*F* = 9.167, *p* = 0.009). No other changes in peak angle data or interactions of sex and time were noted. At initial contact (see Supporting Information S1: supplementary file 3), knee abduction angle was lower at 48 h compared with baseline (*F* = 7.141, *p* = 0.018) and further analysis revealed knee abduction range of motion (from IC to peak knee flexion) was higher (*F* = 13.140, *p* = 0.003) at 48 h. No other changes in angular data at initial contact or interactions of sex and time were noted. Individual changes for peak knee internal rotation angle and knee abduction range of motion are presented in Figure 1.

**TABLE 2:** Peak joint angular data in males and females during a 45° side cut before and 48 h after the multidirectional trial.

	Baseline (°) (*n* = 16)	48 h (°) (*n* = 16)	*d*; ± 95% CI	*p* value from two‐way ANOVA	
				Time	Sex × time
Hip extension (0° = full extension)	31.3 ± 12.0	28.3 ± 14.1	−0.24; ± 0.43	0.252	0.222
Hip adduction (0° = full adduction)	6.6 ± 6.0	6.7 ± 4.4	0.01; ± 0.42	0.948	0.587
Hip internal rotation	10.2 ± 7.6	8.3 ± 7.9	0.24; ± 0.37	0.199	0.800
Knee extension (0° = full extension)	15.6 ± 5.9	15.1 ± 5.0	−0.09; ± 0.34	0.578	0.545
Knee abduction	9.8 ± 4.2	10.1 ± 4.4	0.06; ± 0.36	0.749	0.541
Knee internal rotation	8.9 ± 6.4	11.9 ± 5.0	0.44; ± 0.30	0.009^a^	0.583

Abbreviations: CI = confidence interval; d = effect size.

^a^ indicates a difference between baseline to 48 h.

### 3.2 Kinetics

**FIGURE 2:**
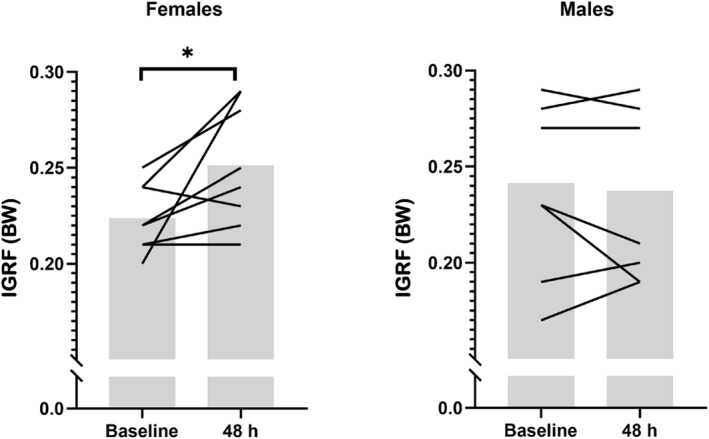
Mean and individual IGRF during the weight acceptance phase in females and males at baseline and 48 h. *indicates a significant sex × time interaction.

Joint moment data before and 48 h after multidirectional running are presented in Table 3 and sex specific joint moment data are provided in Supporting Information S1: supplementary file 4. At 48 h, peak knee extensor moment was lower (*F* = 23.176, *p* < 0.001) and the hip‐knee extensor ratio moment was higher (*F* = 6.917, *p* = 0.020). A small increase in peak hip extensor moment (*F* = 3.988, *p* = 0.066) and peak knee external rotation moment (*F* = 4.122, *p* = 0.062) was observed at 48 h, however this was not significant. No other changes in peak hip and knee moments during the weight acceptance phase of stance were observed. An interaction of sex and time was noted for IGRF (*F* = 5.329, *p* = 0.037). Specifically, IGRF was higher in females at 48 h compared with baseline (*d*; ± 95% CI: 1.4; ± 1.4) but demonstrated a trivial change in males (0.08; ± 0.33) (see Figure 2). Individual changes for peak knee extensor moment and the hip‐knee extensor ratio are presented in Figure 1.

**TABLE 3:** Peak joint moment data during a 45° side cut before and 48 h after the multidirectional trial.

	Baseline (N·m·kg^−1^) (*n* = 16)	48 h (N·m·kg^−1^) (*n* = 16)	*d*; ± 95% CI	*p* value from two‐way ANOVA	
				Time	Sex × time
Hip extension	4.51 ± 1.68	5.01 ± 2.29	0.28; ± 0.29	0.066	0.946
Hip adduction	0.50 ± 0.47	0.64 ± 0.61	0.30; ± 0.68	0.374	0.561
Hip external rotation	0.38 ± 0.21	0.30 ± 0.16	0.37; ± 0.48	0.135	0.863
Knee extension	3.29 ± 0.85	2.90 ± 0.88	−0.43; ± 0.20	0.001^a^	0.170
Knee adduction	1.12 ± 0.56	1.03 ± 0.57	−0.16; ± 0.48	0.513	0.872
Knee external rotation	0.16 ± 0.10	0.22 ± 0.11	0.57; ± 0.58	0.062	0.428
Hip‐knee extensor ratio	1.63 ± 1.22	2.20 ± 1.89	0.44; ± 0.37	0.020^a^	0.155

Abbreviations: CI = confidence interval; d = effect size.

^a^ indicates a difference between baseline to 48 h.

## 4 Discussion

This study's purpose was to assess the effect of EIMD on task achievement and biomechanics of side cuts in males and females. The large increase in CK concentration (∼180%–330%), perceived muscle soreness (∼580%–900%) and moderate increase in sprint times (∼6%–8%) for both males and females provide indirect evidence that EIMD was present 48 h after multidirectional running (Howatson and Milak 2009). These data also reaffirm the occurrence of EIMD after team sport activity, with the magnitude of change in EIMD markers similar to those reported previously after team sport activity (Nedelec et al. 2014). The numerous changes of direction in the multidirectional trial, which require high breaking forces and eccentric contractions to rapidly decelerate, is likely to have caused the EIMD response (Howatson and Milak 2009). Specifically, microdamage to the muscle fibre induced by the stretch of high force eccentric actions causes disproportionate lengthening of the sarcomeres, which has been associated with the appearance of sarcoplasmic proteins (e.g., CK) in the blood (Hody et al. 2019). The initial mechanical damage then triggers a cascade of events leading to secondary EIMD, which has been discussed elsewhere (Hody et al. 2019). As the magnitude and time‐course of EIMD symptoms can vary based on the nature, intensity, and duration of exercise (Douglas et al. 2017; Fochi et al. 2016), we illustrate that multidirectional running mimicking the movement demands of team sport activity offers an ecologically valid model to study EIMD.

The similarity in angle, COM velocity and stance time at baseline and 48 h suggests side cut task achievement did not change with EIMD, albeit a small lengthening of the deceleration phase was evident in females. Whilst a recent systematic review demonstrated impairment of change of direction performance with symptoms of EIMD (Harrison et al. 2024), task achievement of a side cut at a sub‐maximal, controlled speed (present study) is not directly comparable to time taken during a maximal change of direction task (Harrison et al. 2024). Despite no change in side cut task achievement, participants had a small increase in peak knee internal rotation angle at 48 h, which is a common mechanism reported during ACL injuries (Koga et al. 2010; Waldén et al. 2015). EIMD could make it difficult to control the lower limb during weight acceptance, which might be due to delayed neural deficits and/or attenuated fibre excitability (Paquette et al. 2017). A reduction in knee proprioception has also been reported with EIMD (Torres et al. 2010), and can alter single leg landing biomechanics (Nagai et al. 2013). Whilst no previous studies have assessed transverse side cut mechanics with EIMD, an increase of 3.5° in knee internal rotation angle has been observed under fatigue (Cortes et al. 2012), which is similar to the 3° change observed in the present study. A reduction in knee abduction angle at initial contact 48 h after multidirectional running was also observed and could be associated with a reduced risk of ACL injury (Hewett et al. 2005). However, peak ACL strain occurs simultaneously with peak knee abduction angle during a simulated landing task (Kiapour et al. 2014), which remained unchanged at 48 h. Furthermore, knee abduction ROM was increased at 48 h which suggests a reduced ability to control frontal plane motion (Jenkins et al. 2017). In agreement with our hypothesis, these data highlight some small impairments to transverse and frontal knee kinematics during a side cut 48 h after multidirectional running. Practitioners should be mindful of the potential increased ACL injury risk in athletes experiencing symptoms of EIMD, particularly when planning multidirectional training requiring frontal and transverse plane control.

Peak knee extensor moment demonstrated a small decrease in participants at 48 h, whilst the hip‐knee extensor ratio demonstrated a small increase. These data indicate participants shifted the extensor demands away from the knee and towards the hip, to control the deceleration phase of the side cut. Whilst an increase in knee extensor moment has previously been reported during a side cut with fatigue (Savage et al. 2018), a 0.49 N·m·kg^−1^ decrease in knee extensor torque during a drop landing with EIMD has been reported (Tsatalas et al. 2021), which is comparable with the 0.39 N·m·kg^−1^ decrease observed in the present study. Quadricep strength has been associated with knee extensor moment (Asaeda et al. 2019), so impaired quadricep function caused by multidirectional running (Oxendale et al. 2023) likely explains the reduction in knee extensor moment observed. This might serve as a protective mechanism to reduce loading of painful tissues (Tsatalas, Giakas, Spyropoulos, Sideris, Kotzamanidis, et al. 2013), in support of our hypothesis. Whilst a reduction in knee extensor moment might seem favourable (Davies et al. 2022), changes in knee extensor moment alone are considered a minor contributing factor to ACL loading (Bakker et al. 2016), and a pattern of an increased hip and decreased knee extensor moment has been observed in individuals with ACL reconstruction compared to the non‐operated leg and healthy controls (Warathanagasame et al. 2023). Practitioners might therefore consider training and recovery strategies to reduce the shift in extensor demands from the knee to the hip. Whilst no other significant changes in joint moments were observed, knee external rotation moment displayed a small increase at 48 h. Internal tibial rotation moments (expressed as an external moment), when combined with knee abduction moments, probably constitutes the greatest risk of a non‐contact ACL injury (Dempsey et al. 2007) as internal tibial rotation moments are strongly correlated to ACL force (r = 0.78) (Navacchia et al. 2019). Future research should explore if knee transverse kinetics change during a side cut with symptoms of EIMD.

The large increase in IGRF observed here in females indicates an increased risk of ACL injury, given that cadaveric mechanical simulations applying IGRF combined with knee valgus moment, anterior shear force, and internal tibial rotation, induces ACL rupture (Bates et al. 2017) and IGRF asymmetries predict knee kinetics asymmetries in patients following ACL reconstruction (Dai et al. 2014). The increase in IGRF might also suggest a greater requirement to decelerate the downward movement of the COM during the weight acceptance phase (Harry et al. 2017), as a greater IGRF magnitude would indicate a greater change in COM momentum. The moderate increase in the percentage of weight acceptance observed in females suggest the greater IGRF was achieved by lengthening the duration of the deceleration phase of the side cut, which is consistent with previous reports (Mulligan et al. 2024). Lower neuromuscular co‐ordination and insufficient strength from the knee muscles after multidirectional running might make it difficult to counter the external flexion moment during weight acceptance as the GRF vector passes posterior to the knee joint axis, causing IGRF to increase. In agreement, a reduced tolerance to impact and a loss of elastic energy potential has been reported with EIMD (Avela and Komi 1998). Taken together, females exhibited a greater IGRF requirement to decelerate the body during a side cut after muscle damaging exercise, which has been associated with an increased ACL injury risk. This suggests female‐specific training considerations might be warranted when symptoms of EIMD are present, particularly for actions involving high deceleration demands, such as a side cut.

This study is not without limitations. This study confirmed the presence of EIMD, however the mechanisms of EIMD symptoms, and the effects on concentric and eccentric joint torque, were not assessed. Future research in this area should comprehensively investigate the effect of EIMD elicited from multidirectional running, which might provide more insight into the altered biomechanics of side cuts. For example, transcranial magnetic stimulation, twitch interpolation and electromyography assessed during maximal voluntary contractions would provide insight into the extent that the central nervous system and peripheral musculature contribute to force decrements and performance changes after multi‐directional running. Although the sample size used was deemed adequate, future research should incorporate a larger sample of males and females to determine if any other EIMD induced sex differences in side cut biomechanics are apparent, and whether transverse moments change with EIMD or not. Whilst the re‐application of reflective markers at 48 h might reduce the between‐day reliability of motion data, the functional method used to define the hip and knee joint centres, improves repeatability (Besier, Sturnieks, et al. 2003b). Finally, the potential effect of menstrual cycle phase (e.g., early follicular vs. mid‐luteal) on female responses to EIMD should be considered in future research. Considering the novelty of the findings, these limitations do not reduce the impact of the results.

## 5 Conclusion

In conclusion, multidirectional running elicited a similar magnitude of EIMD between sexes. Whilst task achievement and sagittal side cut kinematics were not affected by EIMD, some alterations in side cut biomechanics were observed. Specifically, more reliance on hip extensor moment, compared with knee extensor moment was observed to decelerate the body and small increases in knee frontal and transverse motion were evident. Sex specific differences in braking impulse were observed, as females showed an increased effort to decelerate by lengthening the weight acceptance phase. Collectively, these data suggest a less effective deceleration capacity during a side cut in females when experiencing symptoms of EIMD, despite maintaining task achievement. The increase in knee frontal and transverse motion have been previously identified as ACL injury risk factors, suggesting EIMD might indirectly contribute to ACL injuries in males and females, albeit the small changes observed indicate that the overall impact of EIMD on ACL injury risk might be limited. Practitioners should consider the presence of EIMD when assessing ACL injury risk and designing training involving side cutting, particularly during intensified training periods when EIMD might be present. Future research is required to determine the specific mechanism for the alterations observed.

## Author Contributions

All authors contributed to the study conception and design. Material preparation, data collection and analysis were performed by Chelsea Oxendale. The first draft of the manuscript was written by Chelsea Oxendale and all authors reviewed and edited the manuscript and approved the final version.

## Ethics Statement

Ethical approval was granted for this study (1216‐16‐CO‐SES) from the University of Chester Faculty of Medicine and Life Sciences Research Ethics Committee and is detailed in the methods section.

## Conflicts of Interest

The authors declare no conflicts of interest.

## Supporting information


Supporting Information S1

## Data Availability

Collected data are included in the manuscript and sex specific data are provided in the Supporting Information S1. Raw data is available upon reasonable request to the corresponding author.
