# Supplementary material for: The Effect of Exercise‐Induced Muscle Damage on Lower Limb Side Cut Biomechanics and Task Achievement in Male and Female Team Sport Athletes
Source: Eur J Sport Sci. 2025 Aug 30;25(9):e70051. doi: 10.1002/ejsc.70051 (PMC12397987; doi:10.1002/ejsc.70051)
Supplement: Supplementary file 1 — Supporting Information S1 [file EJSC-25-e70051-s001.docx]

**Supplementary file 1**

**Table 1.** In-direct markers of EIMD in males and females before and 48 hours after the multi-directional trial

|  | *Females* | | *Males* | | |
| --- | --- | --- | --- | --- | --- |
|  | Baseline | 48 h | | Baseline | 48 h |
| *Indirect markers of EIMD* | | | | | |
| CK concentration (U^.^L^-1^) | 93.3 ± 38.9 | 308.9 ± 295.7* | | 155.1 ± 47.8 | 282.3 ± 155.4* |
| Perceived soreness (AU) | 0.5 ± 0.8 | 4.6 ± 1.2* | | 0.7 ± 0.7 | 4.1 ± 2.0* |
| 20 m sprint time (s) | 3.4 ± 0.2 | 3.7 ± 0.3* | | 3.1 ± 0.2 | 3.3 ± 0.2* |
| *Side cut task achievement* | | | | | |
| Centre of mass velocity at IC (m^.^s^-1^) | 4.2 ± 0.3 | 4.3 ± 0.2 | | 4.2 ± 0.2 | 4.2 ± 0.2 |
| Centre of mass velocity at TO (m^.^s^-1^) | 3.8 ± 0.3 | 3.7 ± 0.2 | | 3.8 ± 0.3 | 3.8 ± 0.2 |
| Change in centre of mass angle from IC to TO (°) | 21.0 ± 3.4 | 21.3 ± 2.0 | | 25.9 ± 4.6 | 25.1 ± 4.0 |
| Stance time (s) | 0.2 ± 0.03 | 0.21 ± 0.03 | | 0.25 ± 0.04 | 0.25 ± 0.04 |
| Weight acceptance percentage of stance (%) | 56.0 ± 7.2 | 63.1 ± 10.5^a^ | | 47.7 ± 4.2 | 46.4 ± 4.6 |

*IC = initial contact and TO = toe off. * indicates a significant difference at 48 h compared to baseline. ^a^ indicates a sex x time interaction*

**Supplementary file 2**

**Table 2.** Peak joint angular data in males and females during a 45º side cut before and 48 h after the multi-directional trial

|  | *Females (n = 8)* | | | *Males (n = 8)* | | | *P value from two-way ANOVA* | |
| --- | --- | --- | --- | --- | --- | --- | --- | --- |
|  | Baseline (°) | 48 hr (°) | *d*; ±95% CI | Baseline (°) | 48 hr (°) | *d*; ±95% CI | Time | Sex x Time |
| Hip extension (0° = full extension) | 27.5 ± 13.0 | 21.3 ± 13.4 | 0.43; ±0.43 | 35.1 ± 10.5 | 35.3 ± 11.7 | 0.02; ±0.85 | 0.252 | 0.222 |
| Hip adduction (0° = full adduction) | 4.9 ± 6.4 | 5.7 ± 2.3 | 0.11; ±0.57 | 8.4 ± 5.6 | 7.8 ± 5.5 | 0.1; ±0.72 | 0.948 | 0.587 |
| Hip internal rotation | 12.7 ± 8.6 | 11.1 ± 8.0 | 0.16; ±0.34 | 7.7 ± 5.9 | 5.4 ± 7.0 | 0.35; ±0.9 | 0.199 | 0.800 |
| Knee extension (0° = full extension) | 16.1 ± 5.6 | 15.0 ± 6.0 | 0.19; ±0.56 | 15.1 ± 6.5 | 15.2 ± 4.2 | 0.01; ±0.43 | 0.578 | 0.545 |
| Knee abduction | 11.9 ± 4.9 | 11.7 ± 5.2 | 0.04; ±0.61 | 7.6 ± 2.1 | 8.4 ± 2.8 | 0.31; ±0.65 | 0.749 | 0.541 |
| Knee internal rotation | 8.7 ± 6.3 | 12.2 ± 4.4 | 0.5; ±0.39 | 9.2 ± 7.0 | 11.6 ± 5.9 | 0.31; ±0.65 | 0.009* | 0.583 |

** indicates a difference between baseline to 48 h. d = effect size, CI = confidence interval*

**Supplementary file 3**

Angle data at initial contact at baseline and 48 h during a 45º side cut

|  | Baseline (°)  *(n = 16)* | 48 hr (°)  *(n = 16)* | *d*; ±95% CI | *P value from two-way ANOVA* | |
| --- | --- | --- | --- | --- | --- |
|  |  |  |  | Time | Sex x Time |
| Hip flexion | 39.6 ± 8.8 | 37.7 ± 9.3 | -0.21; ±0.59 | 0.469 | 0.499 |
| Hip abduction | 10.3 ± 5.4 | 10.5 ± 4.0 | 0.03; ±0.49 | 0.890 | 0.370 |
| Hip internal rotation | 7.4 ± 9.0 | 5.2 ± 8.0 | 0.23; ±0.41 | 0.250 | 0.355 |
| Knee flexion | 15.5 ± 6.5 | 16.7 ± 5.1 | 0.17; ±0.46 | 0.446 | 0.518 |
| Knee abduction (+) | 2.4 ± 3.8 | -0.04 ± 3.3 | -0.58; ±0.45 | 0.018* | 0.913 |
| Knee abduction ROM | 7.4 ± 4.9 | 10.0 ± 4.5 | 0.51; ±0.30 | 0.003* | 0.433 |
| Knee external rotation | 5.1 ± 7.7 | 4.4 ± 6.1 | -0.09; ±0.49 | 0.677 | 0.067 |

*d = effect size, CI = confidence interval*

**Supplementary file 4**

**Table 3.** Peak joint moment data in males and females during a 45º side cut before and 48 h after the multi-directional trial

|  | *Females (n = 8)* | | | *Males (n = 8)* | | | *P value from two-way ANOVA* | |
| --- | --- | --- | --- | --- | --- | --- | --- | --- |
|  | Baseline (N^.^m^.^kg^-1^) | 48hr (N^.^m^.^kg^-1^) | *d*; ±95% CI | Baseline (N^.^m^.^kg^-1^) | 48 hr (N^.^m^.^kg^-1^) | *d*; ±95% CI | Time | Sex x Time |
| Hip extension | 4.92 ± 2.19 | 5.43 ± 3.03 | 0.21; ±0.41 | 4.11 ± 0.92 | 4.59 ± 1.27 | 0.46; ±0.56 | 0.066 | 0.946 |
| Hip adduction | 0.70 ± 0.46 | 0.95 ± 0.7 | 0.47; ±1.07 | 0.30 ± 0.42 | 0.35 ± 0.35 | 0.11; ±1.13 | 0.374 | 0.561 |
| Hip external rotation | 0.40 ± 0.22 | 0.31 ± 0.14 | -0.35; ±0.69 | 0.36 ± 0.20 | 0.29 ± 0.19 | -0.31; ±0.73 | 0.135 | 0.863 |
| Knee extension | 2.91 ± 1.03 | 2.41 ± 0.95 | -0.43; ±0.25 | 3.66 ± 0.43 | 3.39 ± 0.44 | -0.56; ±0.51 | 0.001* | 0.170 |
| Knee adduction | 1.32 ± 0.64 | 1.25 ± 0.66 | -0.1; ±0.8 | 0.92 ± 0.42 | 0.81 ± 0.36 | -0.25; ±0.60 | 0.513 | 0.872 |
| Knee external rotation | 0.13 ± 0.09 | 0.21 ± 0.14 | 0.78; ±0.90 | 0.19 ± 0.09 | 0.23 ± 0.09 | 0.34; ±0.91 | 0.062 | 0.428 |
| Hip-knee extensor ratio | 2.14 ± 1.61 | 3.02 ± 2.43 | 0.49; ±0.55 | 1.13 ± 0.24 | 1.37 ± 0.42 | 0.89; ±0.87 | 0.020* | 0.155 |
